# Functional and Structural Studies of a Multidomain Alginate Lyase from *Persicobacter* sp. CCB-QB2

**DOI:** 10.1038/s41598-017-13288-1

**Published:** 2017-10-20

**Authors:** Pei-Fang Sim, Go Furusawa, Aik-Hong Teh

**Affiliations:** Centre for Chemical Biology, Universiti Sains Malaysia, 10 Persiaran Bukit Jambul, 11900 Bayan Lepas, Penang Malaysia

## Abstract

AlyQ from *Persicobacter* sp. CCB-QB2 is an alginate lyase with three domains — a carbohydrate-binding domain modestly resembling family 16 carbohydrate-binding module (CBM16), a family 32 CBM (CBM32) domain, and an alginate lyase domain belonging to polysaccharide lyase family 7 (PL7). Although AlyQ can also act on polyguluronate (poly-G) and polymannuronate (poly-M), it is most active on alginate. Studies with truncated AlyQ showed that the CBM32 domain did not contribute to enhancing AlyQ’s activity under the assayed conditions. Nevertheless, it could bind to cleaved but not intact alginate, indicating that the CBM32 domain recognises alginate termini. The crystal structure containing both CBM32 and catalytic domains show that they do not interact with one another. The CBM32 domain contains a conserved Arg that may bind to the carboxyl group of alginate. The catalytic domain, meanwhile, shares a conserved substrate-binding groove, and the presence of two negatively charged Asp residues may dictate substrate specificity especially at subsite +1. As *Persicobacter* sp. CCB-QB2 was unable to utilise alginate, AlyQ may function to help the bacterium degrade cell walls more efficiently.

## Introduction

Alginate is a major component in the cell walls of brown algae, composed of mannuronate (M) and guluronate (G) arranged as 1,4-linked polysaccharides. It is commercially extracted from seaweed and widely used in the food and pharmaceutical industries. Due to its biocompatibility and ease of gelation, alginate has proved a favourable biomaterial as hydrogels for medical applications such as tissue engineering and drug delivery^1^. Alginate oligosaccharides composed of 3–25 monomers, meanwhile, have multiple biological activities such as stimulating the growth of human keratinocytes^2^ and endothelial cells^3^, as well as promoting growth and root elongation in rice and barley^4,5^.

Alginate is degraded by alginate lyases via a *β*-elimination mechanism to yield 4,5-unsaturated sugar at the non-reducing end. Alginate lyases are classified as either endolytic, which cleave the polysaccharide internally, or exolytic, which cleave the polysaccharide from the end to produce monomers, and both types can exhibit poly-MG, poly-G or poly-M specificity. Among the presently classified 23 polysaccharide lyase (PL) families in the CAZy (*C*arbohydrate *A*ctive en*Zy*me) database (http://www.cazy.org/), alginate lyases are found in families PL5, PL6, PL7, PL14, PL15, PL17 and PL18. These families are structurally diverse, with PL7, PL14 and PL18 alginate lyases, for example, sharing a *β*-jelly roll topology^6^. Alginate lyases can play an important role in producing biologically active alginate oligosaccharides for potential therapeutical and biotechnological applications, as well as monosaccharides for biofuel production^7,8^.

*Persicobacter* sp. CCB-QB2, an agarolytic bacterium isolated from seaweed (genus *Ulva*) in a coastal area of Malaysia^9^, was found to encode a PL7 alginate lyase, AlyQ, in its genome. Like many multidomain alginate lyases, AlyQ contains two additional carbohydrate-binding modules (CBMs) at its N terminus. CBMs bind to a wide variety of carbohydrates, and are commonly found appended to polysaccharide degrading enzymes such as xylanases and chitinases to help target complex substrates like insoluble polysaccharides^10^. AlyQ’s first domain moderately resembles family 16 CBM (CBM16), while the second domain belongs to family 32 CBM (CBM32). In order to understand the relationship between these CBMs and the catalytic domain, we have characterized AlyQ’s activity and substrate binding, as well as solved the structure of a truncated version containing the CBM32 and catalytic domains.

## Results and Discussion

### Resequencing of AlyQ yields a soluble alginate lyase

**Figure 1:**
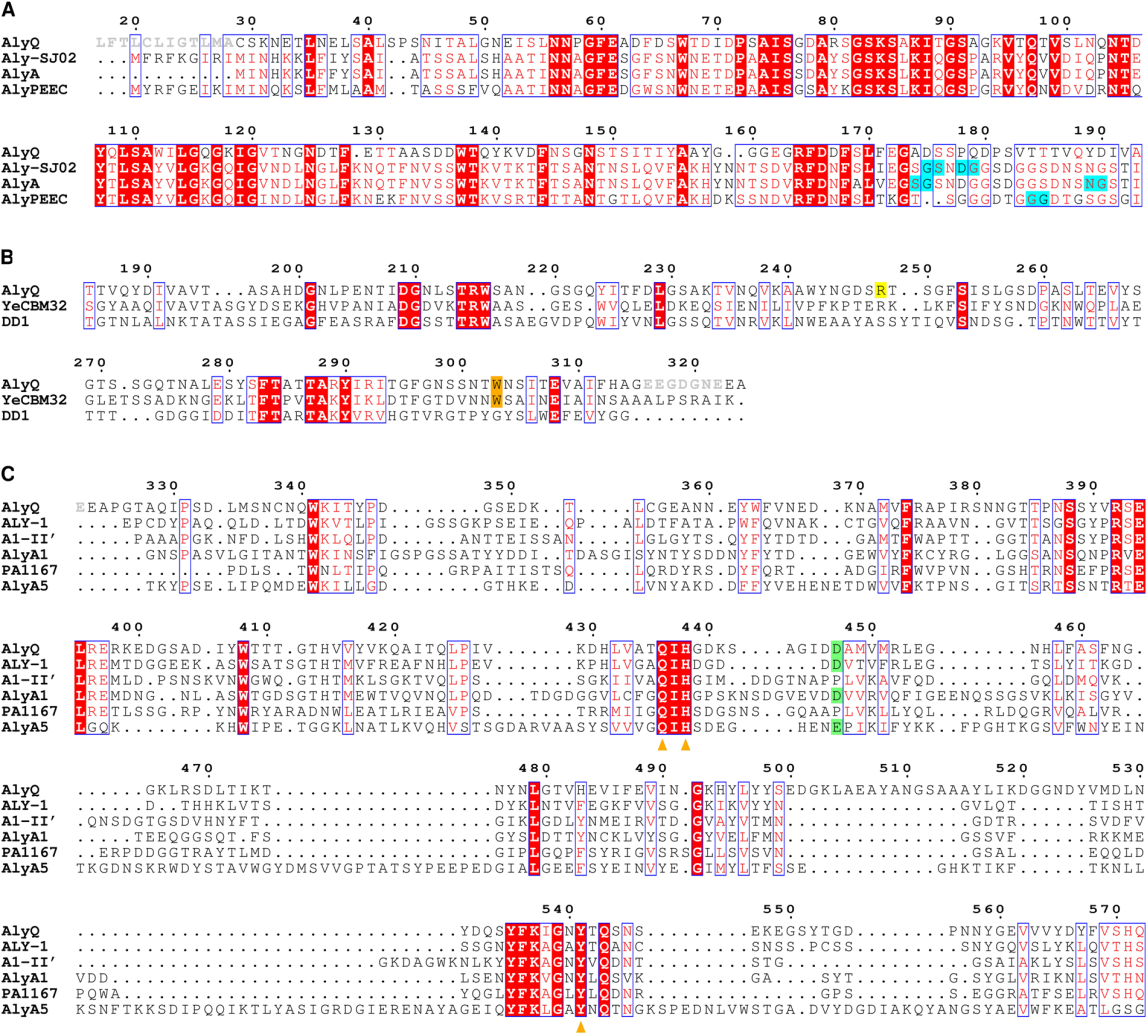
Alignments of AlyQ. (**A**) Domain A, which is reportedly cleaved (cleavage sites in cyan) during enzyme maturation in *Pseudoalteromonas* PL18 alginate lyases aly-SJ02, AlyA and AlyPEEC. AlyQ’s signal peptide is shown in grey. (**B**) Domain B, a CBM32 with the conserved W303 (orange) shown to be critical to the single-domain YeCBM32’s binding to polygalacturonic acid but not conserved in the *Paenibacillus* chitosanase’s DD1 domain. AlyQ’s R248 (yellow) may bind to the sugar’s carboxyl group. The linker between domains B and C is shown in grey. (**C**) Domain C, a PL7 alginate lyase containing the highly conserved Q436, H438 and Y541 (orange triangles) for cleaving the glycosidic bond. PL7 alginate lyases sharing AlyQ’s D447 (green) — *Corynebacterium* ALY-1, and *Z*. *galactanivorans*’ AlyA1_PL7_ and AlyA5 — may constitute a subgroup with a subsite −1 different from that reported in the structures of *Sphingomonas* A1-II′ and *P*. *aeruginosa* PA1167.

AlyQ was cloned initially according to its annotated gene (NCBI WP_053404615) from the genome of *Persicobacter* sp. CCB-QB2, but the expressed enzyme was insoluble. A BLAST search found an almost identical protein from *Persicobacter* sp. JZB09 (WP_060686070), which however contains an extra N-terminal 48 residues. Comparison of both genes revealed a missing cytosine in the *alyQ* gene, whose presence was verified with resequencing. The final gene encodes a protein of 572 residues that also includes the additional 48 residues, sharing 93% identity with the *Persicobacter* sp. JZB09 protein. In addition to the three domains, domains A–C (Fig. 1), it also contains an N-terminal signal peptide sequence (M1–A28) predicted by the SignalP program^11^. The 48 residues included the first twenty residues of domain A, whose absence could have led to the enzyme’s insolubility, and a new construct excluding the signal peptide finally yielded a soluble protein (C29–Q572).

### AlyQ prefers alginate over poly-G and poly-M

**Figure 2:**
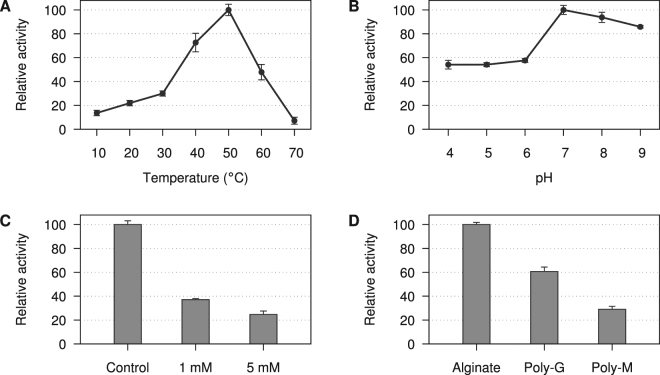
Characterisation of AlyQ’s lyase activity. (**A**) AlyQ was most active at 50 °C. (**B**) The optimum pH was pH 7, and AlyQ experienced a bigger drop in activity at lower pH. (**C**) AlyQ’s activity was reduced to 37.1 and 24.7% in the presence of 1 and 5 mM EDTA. (**D**) AlyQ cleaved alginate most efficiently, and was only 60.7 and 29.0% active on poly-G and poly-M respectively.

AlyQ had the highest activity at 50 °C while it became largely inactivated at 30 °C, retaining only 30% of activity, and below (Fig. 2A). It was also most active at pH 7, and its activity decreased more quickly at lower pH, losing about 40% of activity at pH 4–6 (Fig. 2B). The sudden drop in activity at lower pH compared to that at higher pH could be due to the protonation of the conserved catalytic H438, which decreased its basicity to attack the sugar’s C5 atom. AlyQ was inhibited by EDTA, and its activity was greatly reduced to 37.1% in the presence of 1 mM EDTA (Fig. 2C).

Among the different substrates, AlyQ showed the highest preference for alginate (Fig. 2D). Its activity towards poly-G was 60.7% of that for alginate, but dropped to 29.0% for poly-M. These results indicate that the glycosidic bond between either a GM or MG pair is cleaved most efficiently. The values of AlyQ’s *K*_m_ and *k*_cat_, determined from the Lineweaver–Burke plot, were 2.42 mM and 32.21 s^−1^ respectively (Table 1). While kinetic parameters are normally difficult to compare due to the differences in assay conditions, AlyQ’s *K*_m_ resembles those of several alginate lyases of modest sequence similarity, such as AlyA1_PL7_ (3.087 mM) from *Zobellia galactanivorans* ^12^.

**Table 1:** Kinetic parameters of AlyQ, AlyQ_*BC*_ and AlyQ_*C*_.

	*K*_m_ (mM)	*k*_cat_ (s^−1^)	*k*_cat_/*K*_m_ (mM^−1^s^−1^)
AlyQ	2.42 ± 0.08	32.21 ± 2.00	13.30 ± 0.94
AlyQ_*BC*_	1.46 ± 0.33	10.40 ± 0.68	7.12 ± 1.66
AlyQ_*C*_	1.39 ± 0.56	9.24 ± 1.20	6.64 ± 2.83

### Domain A has an unknown function that does not affect AlyQ’s kinetics

Domain A, a putative CBM sharing modest similarity with CBM16, is found in most PL18 alginate lyases. In several *Pseudoalteromonas* PL18 alginate lyases such as AlyPEEC^13^, AlyA^14^ and aly-SJ02^15^, this CBM16-like domain is cleaved during enzyme maturation to yield only a single catalytic domain. In aly-SJ02, it has further been shown to bind to the catalytic domain when both separate domains were co-expressed in *E*. *coli* cells, suggesting a role in facilitating the folding of the catalytic domain^16^. In contrast, CBM16 domains are reported to facilitate substrate degradation, as deleting the two CBM16 domains of a mannanase from *Caldanaerobius polysaccharolyticus*, Man5A, greatly reduced its activity^17^. The crystal structures of these two CBM16 domains further revealed a binding site that could accommodate a chain of five sugar residues^18^.

In order to investigate the impact of domain A on AlyQ’s activity, a truncated version consisting of only domains B and C, AlyQ_*BC*_, was constructed. The catalytic efficiency (*k*_cat_/*K*_m_) of AlyQ_*BC*_ was not significantly different from that of AlyQ (Table 1), suggesting that, under the tested condition, domain A did not affect AlyQ’s activity. To further investigate the substrate binding activities of domain A, it was expressed alone with the aim of subjecting it to size-exclusion chromatography in the presence of different alginate substrates. However, the construct containing only domain A, AlyQ_*A*_, formed large oligomers which eluted in the void volume, compromising the analysis of the substrate binding results.

The CBM16-like domain of aly-SJ02 which shares an identity of 34% with AlyQ’s domain A, in comparison, did not seem to form large oligomers^16^. Its cleavage site, which is different among the PL18 alginate lyases but always contains at least one Gly residue, is also not conserved in AlyQ (Fig. 1A), which was expressed and purified with domain A intact. AlyQ_*BC*_ also seemed to be expressed normally despite a slightly lower *k*_cat_ value. Furthermore, many PL7 alginate lyases do not contain domain A, such as the one from *Gilvimarinus chinensis* (NCBI WP_020210543) which instead contains two CBM32 domains and shares 61% identity with AlyQ_*BC*_, the characterized AlyPI from *Pseudoalteromonas* sp. CY24 which contains only a single CBM32^19^, as well as the single-domain alginate lyase from *Vibrio variabilis* (GenBank GAL28632) which is 70% identical to domain C, suggesting that domain A may not necessarily be required for the proper folding of the catalytic domain.

As at present the ability of these CBM16-like domains to bind any carbohydrate has not been reported, it is thus unclear how domain A may facilitate AlyQ’s function. This domain probably may not be cleaved in AlyQ, at least not in the same manner as the PL18 alginate lyases due to the absence of the Gly-containing cleavage sites. It is noted from the crystal structure of AlyQ_*BC*_ (discussed below) that domain A may be placed in close proximity to the catalytic domain C, hence interactions between the two may not be impossible.

### The CBM32 domain binds to cleaved alginate

Domain B of AlyQ belongs to CBM32, one of the most diverse CBM families whose members are found to bind a variety of ligands including rigid cell wall polysaccharides and complex glycans^20^. The single-domain YeCBM32 from *Yersinia enterolitica*, 36% identical to domain B, has been shown to bind to polygalacturonic acid^21^, whereas the two CBM32 domains from a *Paenibacillus* chitosanase, DD1 and DD2 of 29–32% identity with domain B, bind specifically to chitosan^22^. To study domain B’s function, a truncated version containing only the catalytic domain, AlyQ_*C*_, was further constructed. However, its *K*_m_ and *k*_cat_ values were very similar to those of AlyQ_*BC*_ (Table 1), indicating that domain B did not seem to facilitate the lyase activity of domain C under the assayed conditions.

**Figure 3:**
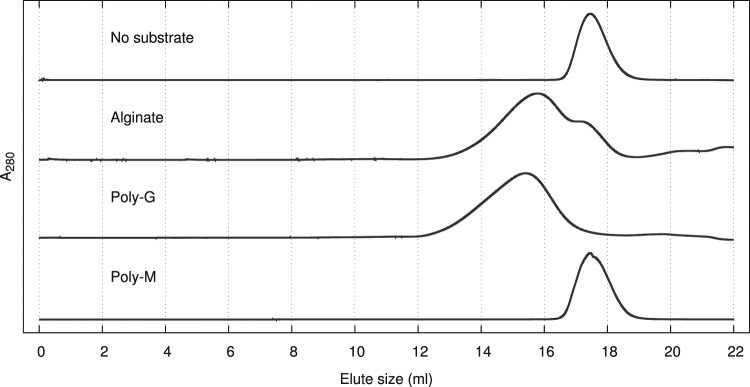
Substrate binding of AlyQ_*B*_ with size-exclusion chromatography. After treatment with minute amounts of AlyQ_*C*_, a peak shift was observed for AlyQ_*B*_ mixed with either alginate or poly-G, but not with poly-M.

To further investigate domain B’s substrate binding ability, AlyQ_*B*_, which contained only domain B, was constructed. When mixed with intact alginate, AlyQ_*B*_ initially was unable to bind to alginate and eluted as a monomer during size-exclusion chromatography. Filtration of the mixture before chromatography was not expected to trap alginate, and the presence of alginate was verified with an increase in absorbance at 235 nm when the filtered mixture was mixed with AlyQ_*C*_. Nevertheless, the experiment was repeated by incubating alginate with minute amounts of AlyQ_*C*_ prior to mixing with AlyQ_*B*_, and a significant shift from the monomer’s peak was observed (Fig. 3). Substituting alginate with poly-G treated with AlyQ_*C*_ also yielded a similar peak shift, but no peak shift was observed with AlyQ_*C*_-treated poly-M. These results show that the CBM32 domain of AlyQ indeed can bind alginate.

CBM32 domains are important for catalysis in many carbohydrate-degrading enzymes such as *α*-*N*-acetylglucosaminidase from *Clostridium perfringens*, AgnC, whose activity on mucin was greatly reduced when three of its six CBM32 domains were truncated^23^. The similar kinetic parameters between AlyQ_*BC*_ and AlyQ_*C*_, meanwhile, could be due to the assayed conditions using soluble alginate, which was easily accessible to AlyQ’s catalytic domain regardless of the presence of the CBM32 domain. Indeed, the AgnC enzyme lacking its three CBM32 domains instead showed increased activity towards a soluble *p*-methoxyphenyl disaccharide, a much simpler substrate than the complex mucin^23^.

The absence of any peak shift with intact alginate in size-exclusion chromatography shows that AlyQ_*B*_ does not bind alginate internally. AlyQ_*B*_’s preference for degraded alginate, on the other hand, indicates that it selectively binds the polysaccharide’s termini, possibly the unsaturated sugar at the non-reducing end generated during catalysis. CBM32 domains binding to terminal carbohydrate have also been reported, for example the structures of AgnC’s CBM32–5 binding a terminal *N*-acetylgalactosamine residue^24^, and the *Paenibacillus* chitosanase’s DD2 binding the non-reducing end of tri-glucosamine^25^. The lack of poly-M binding, meanwhile, might have arisen from the short incubation time with AlyQ_*C*_, whose lower activity towards poly-M was unable to produce sufficient unsaturated alginate termini for AlyQ_*B*_ binding.

**Figure 4:**
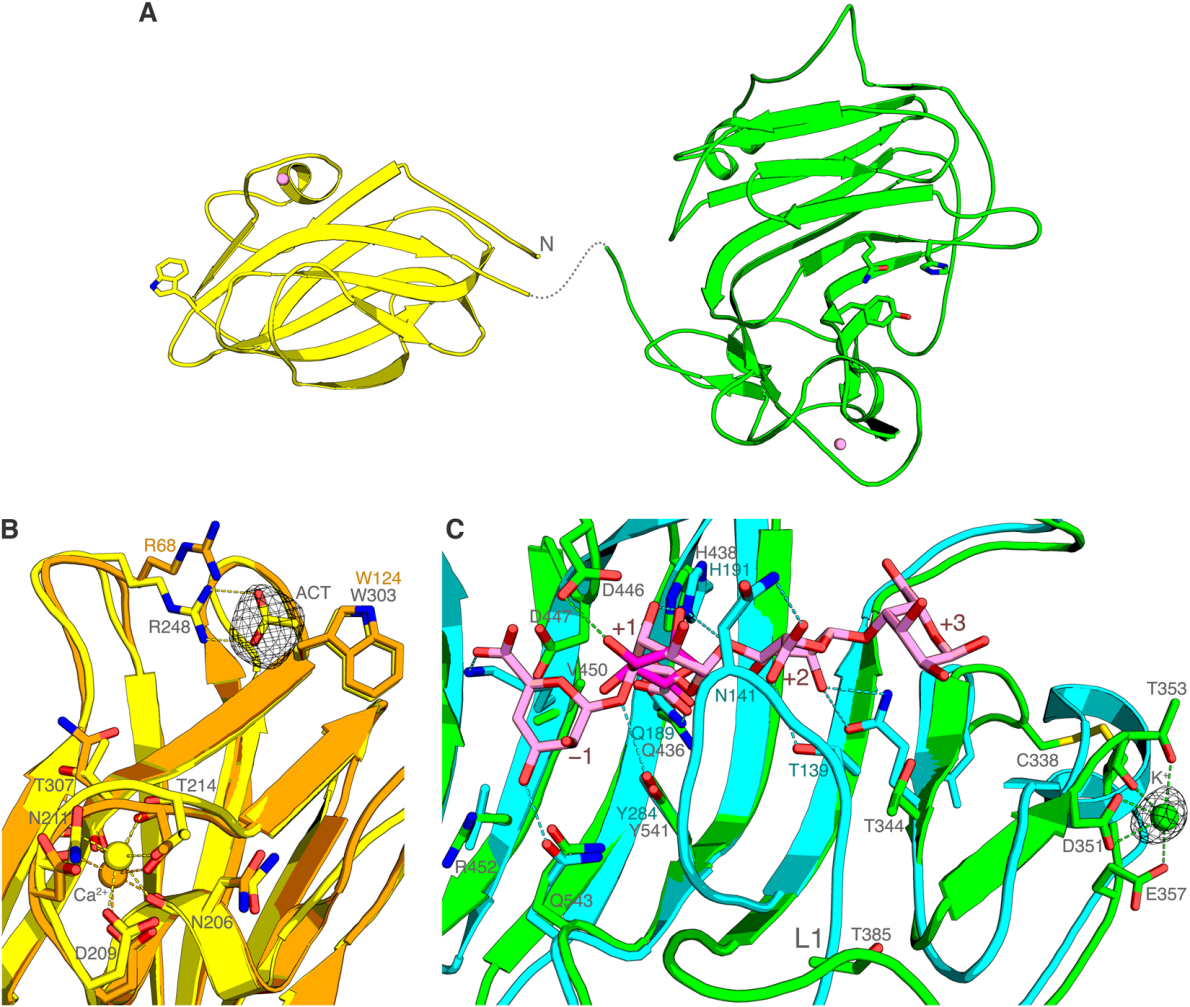
Structure of AlyQ_*BC*_. (**A**) Structure of the CBM32 (yellow) and catalytic (green) domains. While both domains do not interact with each other, domain A, positioned at the N-terminus of the CBM32 domain, may potentially be in close contact with the catalytic domain. Each domain binds a Ca^2+^ or a K^+^ ion (pink spheres). (**B**) Superposition of the CBM32 domain with YeCBM32 (orange). A molecule of acetic acid (ACT) is found binding to R248. The conserved fold between the two enzymes may be specific for binding negatively charged uronic acids. (**C**) Superposition of the catalytic domain with A1-II′ (cyan) and the GGMG ligand (pink) from an A1-II′ complex. While subsites +1 to +3 are comparatively conserved between the two enzymes, repulsion from AlyQ’s D447 may cause the sugar’s carboxyl group at subsite −1 to flip to bind to R452 and Q543. A mannuronic acid (magenta) superposed at subsite +1, meanwhile, does not crash with D446. The *F*_O_ − *F*_C_ omit map is contoured at 2.5 *σ* for the acetic acid and 4 *σ* for the K^+^ ion.

In order to study AlyQ’s mechanism, both full-length AlyQ and the truncated AlyQ_*BC*_ were crystallised. However, only AlyQ_*BC*_ successfully formed crystals, diffracting up to 2.2 Å in space group *P*4_1_2_1_2 with one molecule in the asymmetric unit. In accordance to size-exclusion chromatography results, analysis with Pisa^26^ showed that all crystal contacts were not significant (Supplementary Fig. S1) — AlyQ_*BC*_ exists as a monomer while domains B and C do not interact with one another (Fig. 4A). Residues E316–N321 in the region linking the two domains were disordered. Domain B shares the highest structural similarity with YeCBM32^21^ (Z score 21.6, rmsd 1.3 Å, 33% identity), and both itsN-terminal and C-terminal ends converged to hydrogen bond each other, potentially placing domain A close to the catalytic domain.

A Ca^2+^ ion is found coordinated by the side chains of the strictly conserved D209 and T214, and the main chains of N206, N211, T214 and T307 (Fig. 4B). This Ca^2+^-binding site is conserved in CBM32 members regardless of their substrate specificity, suggesting a structural role in the maintenance of the fold. W124 of YeCBM32, which when substituted with Ala resulted in only very weak affinity for the ligand polygalaturonic acid^21^, is conserved in domain B as W303, and the region around this Trp, which is highly variable and involved in binding different sugars in CBM32 members, is also highly similar between AlyQ and YeCBM32. Moreover, an acetic acid molecule from the crystallisation buffer is found binding to the nearby R248 (Fig. 4B), which may mimic the carboxyl group of an uronic acid. Indeed, our preliminary studies on an AlyQ_*B*_ complex indicate that domain B binds to the unsaturated sugar.

### The catalytic domain has a different binding mode at subsite −1

Domain C is a functional PL7 alginate lyase on its own. Structurally it is most similar to the alginate lyase from *Corynebacterium* sp., ALY-1 (Z score 29.0, rmsd 1.5 Å, 35% identity)^27^. Residues making up the sugar binding groove are highly conserved in both enzymes. In order to predict the AlyQ’s mechanism of action, a structure of *Sphingomonas* sp. A1 alginate lyase A1-II′ complexed to the GGMG ligand (PDB 2ZAA)^28^, which is also similar to AlyQ (Z score 24.0, rmsd 2.3 Å, 28% identity), was superposed onto domain C (Fig. 4C). Subsites +1 to +3 are relatively well conserved between the two, and the three key residues for catalysis — Q436 neutralizing the substrate’s carboxyl group, H438 as a general base and Y541 as a general acid — were superposed well with A1-II’s Q189, H191 and Y284 to interact with the ligand, indicating AlyQ also employs a similar mechanism for alginate cleavage.

Subsites +1 and +2 in A1-II′ are also coordinated by a conserved loop, L1, whose flexibility has been shown by mutagenesis to be essential for substrate binding and product releasing^28^. A shift in AlyQ’s loop L1, however, is observed, which may represent the open conformation (Fig. 4C). Likewise, in the structures of several PL7 alginate structures their loop L1 also resembles the open conformation. By modelling AlyQ’s loop L1 into the closed conformation of A1-II′, AlyQ’s T385 as well as N387 could be similarly positioned into binding the ligand at subsites +1 and +2 as T139 and N141 of A1-II′. The conservation of AlyQ’s T385 as Thr or Ser in these alginate lyases (Fig. 1C), so as to bind to the sugar’s carboxyl group at subsite +2 in the closed conformation, indicates that loop L1 may represent a functionally important feature of PL7 alginate lyases.

Subsite −1 of AlyQ, in contrast, is totally different from that of A1-II′ except Q543. Notably, if the GGMG ligand were to bind to AlyQ as it did to A1-II′, it would have crashed with AlyQ’s D446 and D447 at subsites +1 and −1 — D447 would especially repel the carboxyl group at subsite −1. In fact, among the presently known PL7 alginate lyase structures, only PA1167 from *Pseudomonas aeruginosa* ^29^ (PDB 1VAV) shares the same conformation of subsite −1 with A1-II′, while the rest contain either an Asp^12,27^ or Glu^12^ similar to AlyQ’s D447 (Fig. 1C). It is thus obvious that these PL7 alginate lyases recognise subsite −1 differently — due to repulsion from the Asp/Glu, the carboxyl group of an uronic acid may possibly flip to bind to, in AlyQ’s case, R452 and Q543. Subsites −1 and +1 are expected to bind preferably to either a GM or MG dimer as both poly-G and poly-M are cleaved less efficiently, and D446 seems to accommodate a superposed mannuronic acid better than a guluronic acid at subsite +1 (Fig. 4C).

The catalytic domain of AlyQ is also found to bind an ion coordinated by the side chains of D351, T353 and E357 (Fig. 4C), which is not observed in the structure of ALY-1 (PDB 1UAI) or any other PL7 alginate lyases. As AlyQ_*C*_’s activity was reduced to below 40% in the presence of the chelating agent EDTA (Fig. 2C), it could be tempting to conclude that this ion was a Ca^2+^ ion important for AlyQ’s activity. However, EDTA was also found to reduce the activity of AlyA1_PL7_ by 90%^12^, whose structure did not bind any Ca^2+^ ions but a Na^+^ ion most likely due to crystal packing (PDB 3ZPY). This binding site of AlyQ is also not well conserved, and therefore the possibility that the EDTA effect on AlyQ may be due to other factors, for example Ca^2+^-assisted substrate binding to neutralise the carboxyl group as observed in certain PL families^6^, cannot be ruled out. In the absence of strong evidence, this ion was modelled as a K^+^ ion derived from the crystallisation buffer. Intriguingly, C355 from this loop and C338 form a disulphide bond which, again, is not conserved in the other alginate lyase structures.

### AlyQ may help break down cell walls

*Persicobacter* sp. CCB-QB2 is an agarolytic bacterium that possibly feed on seaweed. Although it can degrade alginate and agar, the bacterium does not seem to be able to utilise them as it could not grow when supplemented with only either one. In the bacterial alginate metabolic pathway, alginate is first depolymerized into oligosaccharides by endolytic alginate lyases, then further degraded into unsaturated monosaccharides by exolytic alginate lyases. These monosaccharides are spontaneously converted into 4-deoxy-L-erythro-5-hexoseulose uronic acid (DEH), which is then converted into 2-keto-3-deoxy-D-gluconic acid (KDG) by KDG reductase^30^. *Persicobater* sp. CCB-QB2’s genome, however, does not encode an exolytic alginate lyase that plays the critical role in producing DEH.

While *Persicobacter* sp. CCB-QB2 does contain many key enzymes for agarose metabolism, which may explain its agarolytic activity, and displayed a diauxic growth behaviour when supplemented with tryptone and agar as nitrogen and carbon sources^9^, the cells could have prioritized tryptone metabolism over agar metabolism during the diauxic growth. These polysaccharide-degrading enzymes including AlyQ, therefore, may likely function to help the bacterium degrade the structurally complex cell walls of red and brown algae for nutrient acquisition. By anchoring the extracellular enzyme around cleaved alginate, AlyQ’s CBM32 domain may help concentrate the enzyme on enlarging a single cleaved site instead of the enzyme spreading over a large area. Alternatively, AlyQ may function to degrade other polysaccharides that contain certain uronic acids, allowing the bacterium to utilise them as a carbon source.

## Methods

### Cloning, protein expression and purification

The full length of AlyQ, excluding its N-terminal signal peptide (C29–Q572), was cloned into the pCold III vector (TaKaRa) between the *Nde* I and *Hin*d III sites, with a six-His tag inserted before the stop codon. The ligated vector was then transformed into *Escherichia coli* BL21(DE3) cells and verified by DNA sequencing. The truncated enzymes, AlyQ_*BC*_, AlyQ_*A*_, AlyQ_*B*_ and AlyQ_*C*_, were similarly constructed.

Cells were grown in 1 L of LB broth supplemented with 100 µg/ml ampicillin at 37 °C. The culture was cooled to 15 °C when the OD_600_ reached 0.6, and isopropyl-*β*-thiogalactopyranoside (IPTG) was added to a final concentration of 0.1 mM. After 16-hour incubation, the cells were harvested by centrifugation, resuspended in 50 mM Tris–HCl, 100 mM NaCl, pH 8.0, sonicated and centrifuged again to remove cell debris.

The supernatant was first applied to a Ni^2+^-charged HiTrap IMAC HP column (GE Healthcare), and eluted with a gradient of 0–1 M imidazole. The protein was then further purified by size-exclusion chromatography using a Superdex 200 column (GE Healthcare). Protein purify was assessed on 10% SDS-PAGE gels. All other truncated enzymes were similarly purified.

### Synthesis of alginate substrates

Poly-M, poly-G and poly-MG alginate blocks were prepared by partial acid hydrolysis of alginate^31^. Briefly, 50 g of sodium alginate (Sigma) was partially hydrolysed in 1 L of 0.3 M HCl at 100 °C for 20 minutes. After centrifugation, the supernatant containing poly-MG was neutralised with NaOH and precipitated with ethanol. The insoluble precipitate was further hydrolysed with 1 L of 0.3 M HCl for 24 hours, centrifuged and dissolved with 1 M NaOH. After adjusting the pH to 2.85 and centrifugation, the precipitate containing poly-G was dissolved in 10 mM NaOH while the supernatant containing poly-M was neutralized with 0.1 M NaOH, and both were precipitated with ethanol.

Purity of the synthesized alginate blocks were verified with circular dichroism analysis, with the poly-M’s mannuronate content estimated to be 90% and poly-G’s guluronate content 80%. The mannuronate content of sodium alginate was about 60%.

### Enzyme assays

All measurements were performed in triplicate and the mean values were calculated. AlyQ activity was determined by measuring continuously the increase in absorbance at 235 nm, which is due to the formation of the 4,5-unsaturated bond at the non-reducing end. Reactions were carried out at room temperature with 1 mg/ml alginate in 20 mM Tris–HCl, 500 mM NaCl, pH 7. The optimal pH was determined by replacing the buffer with acetate for pH 4–5, MES for pH 6, and Tris–HCl for pH 7–9, whereas the optimal temperature was determined at 10–60 °C with the substrate incubated accordingly for five minutes prior to measurements. Substrate specificity studies were carried out by replacing alginate with either poly-G or poly-M, while EDTA effects were evaluated in the presence of 1 and 5 mM EDTA.

The kinetic parameters were measured by incubating 0.05–3 mg/ml alginate at 30 °C before adding the enzyme to a final concentration of 70 nM. As alginate consists of mannuronic acid and guluronic acid of the same molecular weight, 176 g/mol was assumed for each residue after subtracting 18 g/mol for H_2_O loss during polymerisation. The *K*_m_ and *k*_cat_ values were determined from the Lineweaver–Burk plots, using an extinction coefficient of 6,150 M^−1^ cm^−1^ for measuring the unsaturated product concentration^32^.

### Substrate binding

Substrate binding studies of AlyQ’s CBM32 domain, AlyQ_*B*_, were performed using size-exclusion chromatography. Equal volumes of 10 mg/ml substrate (alginate, poly-M or poly-G), which was either intact or treated beforehand with minute amounts of the catalytic domain, and 3 mg/ml AlyQ_*B*_ were mixed, incubated for 20 minutes, and then applied to the Superdex 200 column.

### Crystallisation and data collection

AlyQ_*BC*_ was crystallised at 10 mg/ml using the sitting-drop vapour diffusion technique at 20 °C. Crystals were formed in 30% PEG 3,350, 0.1 M sodium acetate pH 4.0, and 0.2 M potassium phosphate. Diffraction data for a flashed-cooled crystal at 100 K were collected on a Rigaku MicroMax-007 HF X-ray generator equipped with an R-AXIS IV^++^ area detector, and processed with the Rigaku CrystalClear software in space group *P*4_1_2_1_2.

### Structure determination

The structures of YeCBM32^21^ (PDB 2JDA) and Aly-1^27^ (PDB 1UAI) were used for solving the structures of both domains B and C by molecular replacement with Molrep^33^. The combined structure was then built and refined using REFMAC^34^ and Coot^35^. Data collection and refinement statistics are summarised in Table 2. Structural alignments were performed with the Dali server^36^, manually edited and presented with ESPript^37^. Figures were generated using PyMol (http://www.pymol.org).

**Table 2:** Data collection and refinement statistics for AlyQ_*BC*_.

**Data collection**	
Space group	*P*4_1_2_1_2
Cell dimensions *a*, *b*, *c* (Å)	74.32, 74.32, 179.73
Resolution (Å)	2.30 (2.38–2.30)
Completeness (%)	97.5 (98.4)
Redundancy	2.90 (2.91)
Mean *I*/*σ*(*I*)	5.8 (2.1)
*R*_merge_	0.096 (0.396)
**Refinement**	
No. of unique reflections	21,500
*R*_work_/*R*_free_	0.225/0.278
Rmsd bond lengths (Å)	0.015
Rmsd bond angles (°)	1.715
Mean *B*-factors (Å^2^)	39.43
Ramachandran plot	
Favoured (%)	96.6
Allowed (%)	3.4

Values for the outer shell are given in parentheses.

## Electronic supplementary material

Supplementary Information
