# Supplementary material for: Functional and Structural Studies of a Multidomain Alginate Lyase from Persicobacter sp. CCB-QB2
Source: Sci Rep. 2017 Oct 20;7:13656. doi: 10.1038/s41598-017-13288-1 (PMC5651945; doi:10.1038/s41598-017-13288-1)
Supplement: Supplementary file 1 — Supplementary Information [file 41598_2017_13288_MOESM1_ESM.pdf]

Supplementary Information

Functional and Structural Studies of a Multi-Domain Alginate Lyase from *Persicobacter* sp. CCB-QB2

Pei-Fang Sim, Go Furusawa & Aik-Hong Teh

Centre for Chemical Biology, Universiti Sains Malaysia, 10 Persiaran Bukit Jambul, 11900 Bayan Lepas, Penang, Malaysia

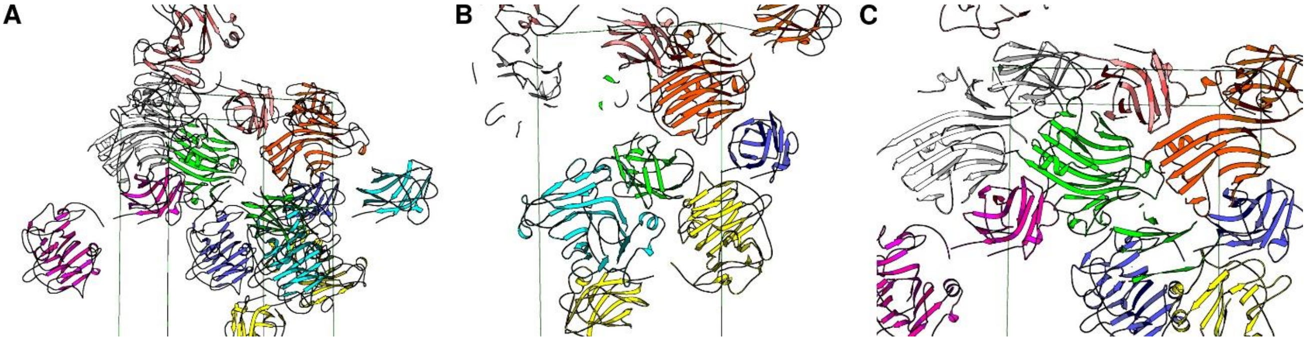

Supplementary Figure S1. Crystal contacts. (**A**) The asymmetric unit contains only a molecule of AlyQ$_{BC}$ (green), which is in contact with a total of seven other molecules. (**B**) A CBM32 domain (green) is surrounded by the catalytic domain of three other molecules (orange, cyan and yellow). (**C**) A catalytic domain (green) in turn makes contact with three CBM32 domains (pink, grey and magenta) as well as one other catalytic domain (purple).
